# Establishing Effective Patient Engagement Through a Terms of Reference to Foster Inclusivity and Empowerment in Research: Example From a Healthcare Transition Quality Indicators Project

**DOI:** 10.1111/hex.70113

**Published:** 2024-11-28

**Authors:** Sarah E. P. Munce, Tomisin John, Dorothy Luong, Sarah Mooney, Lisa Stromquist, Kyle Chambers, Marilyn Crabtree, Sanober Diaz, Gina Dimitropoulos, Megan Henze, Amanda Higgins, Elaine Li, Samadhi Mora Severino, Melanie Penner, Jacklynn Pidduck, Michelle Wan, Laura Williams, Darryl Yates, Alene Toulany

**Affiliations:** ^1^ Bloorview Research Institute Holland Bloorview Kids Rehabilitation Hospital Toronto Ontario Canada; ^2^ Institute of Health Policy, Management and Evaluation University of Toronto Toronto Ontario Canada; ^3^ Rehabilitation Sciences Institute University of Toronto Toronto Ontario Canada; ^4^ Department of Paediatrics, Division of Adolescent Medicine The Hospital for Sick Children Toronto Ontario Canada; ^5^ Child Health Evaluative Sciences SickKids Research Institute Toronto Ontario Canada; ^6^ KITE, Toronto Rehabilitation Institute University Health Network Toronto Ontario Canada; ^7^ Stollery Children's Hospital Alberta Health Services Edmonton Alberta Canada; ^8^ Alberta Strategy for Patient Oriented Research Support Unit University of Calgary Calgary Alberta Canada; ^9^ Integrated Knowledge Translation Panel Member Ottawa Ontario Canada; ^10^ Children's Healthcare Canada Ottawa Ontario Canada; ^11^ Provincial Council for Maternal and Child Health Toronto Ontario Canada; ^12^ University of Calgary Calgary Alberta Canada; ^13^ Surrey Place Toronto Ontario Canada; ^14^ IWK Health Halifax Nova Scotia Canada; ^15^ School of Health Policy and Management York University Toronto Ontario Canada; ^16^ Autism Research Centre Holland Bloorview Kids Rehabilitation Hospital Toronto Ontario Canada; ^17^ University Health Network Toronto Ontario Canada; ^18^ Garry Hurvitz Centre for Brain & Mental Health The Hospital for Sick Children Toronto Ontario Canada; ^19^ Department of Paediatrics, Temerty Faculty of Medicine University of Toronto Toronto Ontario Canada

**Keywords:** integrated knowledge translation, knowledge user involvement, patient engagement, terms of reference

## Abstract

**Introduction:**

Patient engagement in research aims to foster meaningful partnerships, integrating patient experiences into the research process. This paper describes the development of a Terms of Reference (ToR) to support these meaningful partnerships. While engagement improves data collection and empowerment, ineffective engagement can lead to negative outcomes. A well‐developed ToR promotes a structured, inclusive, and respectful process.

**Methods:**

Using an integrated knowledge translation (iKT) approach, we established a panel of youth, caregivers, healthcare providers, and healthcare leaders/decision‐makers. Through collaborative discussions, we incorporated key elements into the ToR, including values, roles, decision‐making processes, and recognition of contributions.

**Results:**

To promote effective engagement the ToR included sections to encourage open, transparent and vulnerable dialogue, evaluation, and accommodations for disabilities. The ToR draft was reviewed and refined by panel members for clarity. Regular reviews and updates will keep the ToR a living document and adaptable to the evolving engagement process.

**Conclusion:**

The implementation of our ToR is designed to foster inclusivity, mutual respect, and accountability, avoiding tokenistic partnership, enriching the experience for patients and researchers alike, and ultimately enhancing research quality.

## 1 Introduction

Patient engagement in research involves a partnership between researchers, patients, and oftentimes, other knowledge users [1]. The Canadian Institute for Health Research Strategy for Patient‐Oriented Research (CIHR‐SPOR) defines patient engagement as the “meaningful and active collaboration in governance, priority setting, conducting research and knowledge translation” [1]. This approach shifts the role of patients from being simply study participants to collaborative members or partners, integrating their experiences and expertise in the research process more fully. Similarly, patient contributions can range from consultative roles to full research team membership. The goal of adopting a patient engagement approach is to guide the research process and shape the results to align directly with the needs and priorities of those it is intended to benefit [1]. The literature highlights numerous benefits that patient engagement offers to both patients and researchers. For researchers, patient engagement can enhance data collection and dissemination efforts, build rapport with communities, and improve research effectiveness [2]. For patients, it can foster an increased sense of voice, value, and the acquisition of life skills [2].

Despite these benefits and its growing use in research, patient engagement is often done inappropriately. This can have negative consequences, such as patients feeling that their contributions are tokenistic, their lived experiences devalued, and that they are not appropriately supported to participate in ways they would have preferred [3]. Furthermore, perceived power imbalances, including the sharing of vulnerable information and the failure to acknowledge and address these imbalances, can lead to negative and even traumatic experiences for patient partners [3]. As a result, such experiences can drive patient partners and others in their network away from future involvement due to the consequences of trauma. These challenges highlight the importance of best practices for research teams implementing patient engagement approaches. Regardless of the patient's role and level of involvement, establishing and adhering to guiding principles can ensure that patient engagement is meaningful, authentic, and empowering.

Numerous models and frameworks outline best practices for engaging knowledge users, including patients, and implementing effective engagement activities. An example is the SPOR Patient Engagement framework, which emphasises inclusiveness, support, mutual respect, and co‐building as guiding principles for integrating patient engagement into research [1]. By adhering to these models and frameworks, researchers can create a collaborative environment that meets the study's and knowledge users' needs. For example, a Participatory Action Research Approach study presented a 10‐step framework to promote culturally inclusive and appropriate community engagement in Aboriginal health research [4]. As part of their patient engagement efforts, the authors collaborated with members of an Aboriginal community through a reference group and co‐created a terms of reference (ToR), demonstrating an effective approach to building and maintaining connections between research teams and the public [4]. Developing a ToR provides research teams with an opportunity to tailor guiding principles and core considerations in their engagement approach [5].

### 1.1 What Is a Terms of Reference?

A ToR defines the collaborative nature of a group working together to accomplish a common goal [6]. It is a key step for generating inclusivity, accountability, support, and shared expectations for all members throughout the engagement process [6]. When developing a research study that includes patient engagement, it is crucial that research teams develop a structure for engagement practices within the study's ongoing activities [7]. To support patient engagement in health research, governance structures are commonly established in the form of steering committees, advisory councils, engagement panels or in some cases, engagement units within an organisation [1, 7]. Also known as a charter or governance plan, a ToR should include the engagement group's purpose, structure, roles and responsibilities, and be easy to understand [6]. Additionally, it should be co‐created by all members of the group, including patients and other knowledge users and be modifiable to reflect the ongoing collaborative process [6].

### 1.2 Developing a Terms of Reference

We applied a patient engagement approach in an ongoing research study focused on identifying quality indicators for the transition of youth from paediatric to adult care services [8]. Our study uses an integrated knowledge translation (iKT) approach, whereby knowledge users are engaged throughout the research process. An iKT panel consisting of youth, caregivers, healthcare providers, and health system leaders with lived experiences in healthcare transitions was established at the study's onset. The panel members are actively involved in key decisions, ensuring that the study is comprehensive and reflective of the perspectives of youth and their families. In an inaugural iKT panel meeting, the research team together with the iKT panel lead, invited panel members to share their insights and perspectives on existing patient engagement frameworks and approaches that could shape the direction of our project. Recognising the panel's expertise and experience, we ensured that all members had the opportunity to provide their feedback. Through open dialogue, elements from various existing patient engagement frameworks were identified as important to incorporate. The next step involved implementing these recommendations into a ToR that would serve as a guide for the engagement process.

When designing the ToR, we drew inspiration from commonly used domains seen in existing collaborative terms of reference documents specifically tailored for engaging patients within and beyond the research environment. We were especially mindful of youth knowledge users and best practices and strategies for engaging them in ways that would empower them [9]. To build a meaningful partnership for effective collaboration with the iKT panel members, eight core areas were initially identified by the research team using existing models and frameworks for iKT and feedback from the panel members. These include providing background information and outlining the membership; values and principles of the engagement approach; clearly defined roles, responsibilities, and opportunities; an established decision‐making process; an outline of the expected outputs; and recognition of the contributions and potential benefits for panel members. To acknowledge and show respect for the unique experiences of all individuals and promote inclusivity, an additional section called “creating brave spaces” was added to the ToR. This section encourages an environment where everyone feels comfortable sharing their thoughts and ideas. The group determined that the ToR should also include an acknowledgement statement to demonstrate a commitment to embracing and upholding acceptance of all differences within their community. For this research study, we included the following acknowledgement statement ‐ *“We acknowledge that we are diverse individuals with different social identities, and we are committed to holding the highest standards of respect and creating a safe space for all in their interactions and discussions at panel meetings.”*

**Figure 1:**
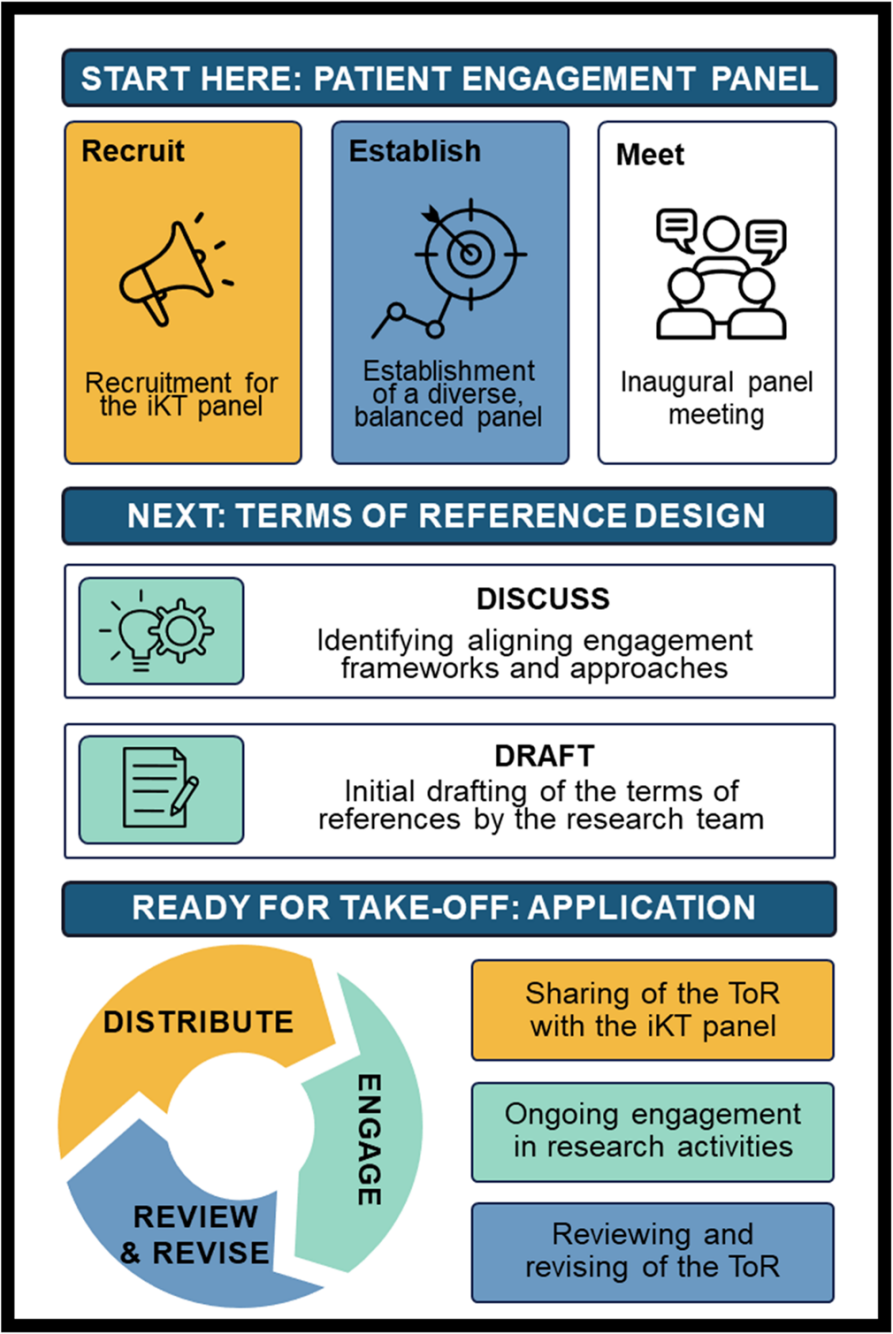
Design process for terms of reference.

A section on evaluation was also included to outline how to measure the impact of the engagement process on all aspects of the study, the panel members, and the ToR as a tool for engagement. Considering the research topic aimed at supporting youth with chronic health conditions, a panel member suggested including a section that explicitly outlines the available accommodations for people with disabilities. This helped to ensure all panel members were supported to actively participate in the engagement process, see Figure 1. For our design process and Supporting Information: Appendix I. For the final sub‐headings included in our ToR and a description of the information.

The first draft was created by the research team and distributed to the panel members via email. Panel members were invited to review, comment and make edits to the structure, wording and content of the ToR. Questions were included within the draft to promote additional input on areas where the research team felt the panel members' valuable and constructive input could further guide its design. Suggestions were made to modify specific words and sentences to make them clearer, more appropriate, and in some cases more inclusive. For example, instead of using “physical ability” the term “dis/ability or illness” was recommended. Similarly, the words “youth and family‐friendly summary” were deemed more appropriate than the term “lay summary.” Panel members also emphasised the necessity of being intentional and clear on the engagement processes outlined in the ToR. One member proposed the inclusion of “specific periods for [the document] review, while still allowing for flexibility for changes to be made at any point needed.” Regarding the decision‐making process outlined in the ToR, it was recommended that an action plan be included to address potential conflicts that might arise when decisions are not consistent with the established values and principles or due to differing perspectives. As the ToR is a living document, updates will be made regularly to align with the evolution of the engagement process and revised versions will be circulated to panel members for approval. This technique will continue throughout the research study period.

### 1.3 Key Considerations

ToRs should be bidirectional, where the benefits to and rights of members are clearly defined. Establishing guiding values and principles that apply to all parties in the relationship is one way to do this. Explicit examples are needed to highlight how these values and principles are bidirectional in nature and are expected to be implemented, not merely suggestions. As part of our commitment to transparency, we have specified several perceived benefits within the ToR for the iKT panel, including authorship recognition, compensation for time, and access to training and leadership opportunities to build capacity in patient partners.

Once developed, ToRs should remain living documents, periodically reviewed and adjusted as needed throughout the engagement and study process to reflect the dynamic nature of relationships. This is particularly salient for longer‐term studies where engagement activities can change depending on the stage of research. Feedback should be obtained in a meaningful way, and considerations around flexibility and ease of providing feedback should be implemented (see Supporting Information: Appendix II. Feedback Checklist for key tips on obtaining feedback).

## 2 Conclusion

Diminishing power dynamics between researchers and knowledge users is fundamental to achieving meaningful patient engagement and avoiding the negative consequences of poorly executed patient engagement [10]. Establishing ToRs for engagement relationships is a crucial step in this process. A well‐defined ToR should outline clear, bidirectional values and principles, define roles and responsibilities, and incorporate feedback from all parties, thereby fostering an environment of inclusivity, mutual respect, and accountability. When effectively implemented, ToRs enable research teams to mitigate common challenges to obtaining meaningful engagement and create truly equitable partnerships. It is critical that all parties regularly review and update the ToR to ensure it remains a living document, adaptable to the evolving needs of the study and its members. This approach not only enhances the quality and relevance of the research but recognises and amplifies the invaluable contributions of patients and other knowledge users, ensuring their voices are heard and respected throughout the research journey.

## Author Contributions

Sarah E. P. Munce, Tomisin John, Dorothy Luong, and Alene Toulany wrote the editorial, and all other authors critically reviewed and edited for content. Sarah E. P. Munce, Alene Toulany, Sarah Mooney, and Lisa Stromquist are co‐investigators of the quality indicators for transition to adult care study. Sarah E. P. Munce and Sarah Mooney co‐lead the quality indicators for transition integrated knowledge translation (iKT) panel. All authors approved the final version of this editorial.

## Conflicts of Interest

The authors declare no conflicts of interest.

## Supporting information

Supporting information.

## Data Availability

The authors have nothing to report.
