# Supplementary material for: Establishing Effective Patient Engagement Through a Terms of Reference to Foster Inclusivity and Empowerment in Research: Example From a Healthcare Transition Quality Indicators Project
Source: Health Expect. 2024 Nov 28;27(6):e70113. doi: 10.1111/hex.70113 (PMC11604592; doi:10.1111/hex.70113)
Supplement: Supplementary file 1 — Supporting information. [file HEX-27-e70113-s001.docx]

**Establishing Effective Patient Engagement Through a Terms of Reference to Foster Inclusivity and Empowerment in Research: Example from a Healthcare Transition Quality Indicators Project**

**APPENDIX**

**Appendix I. Terms of Reference Structure and Description**

| **Section** | **Description** |
| --- | --- |
| **Title** | - Include the project title and engagement group name |
| **Background and Membership** | - The project background information and activities. - The purpose and composition of the patient engagement group and membership type, if applicable. - Other additional information to be included are key definitions, the engagement approaches and frameworks to be followed, and the purpose of the ToR. |
| **Values and Principles** | - List the fundamental ideals (values) agreed upon for the engagement process and their descriptions (principles). - With each value and principle, include some additional context and examples of how they are *applied* in practice. - Other included additional information could be the underlying framework of these values and principles. |
| **Creation of Brave Spaces** | - The ‘creation of brave spaces’ section explicitly promotes inclusivity and promotes a safe space for all members to express themselves without fear of judgment or discrimination. - The inclusion of an acknowledgement statement as a declaration of a commitment to the acceptance of the diversity of all members can further drive the message. |
| **Accommodations** | - Include accommodation provided to support active engagement in the study activities. |
| **Roles, Responsibilities and Opportunities** | - Outline the specific tasks as part of the engagement process. - Distinguish the roles of the project team and the patient engagement group. - If applicable, outline the required expectations, responsibilities, and additional, optional opportunities. - Provide examples for all activities within the project process by listing specific tasks for context. |
| **Decision Making Process** | - The decision-making process should clearly outline the steps and factors involved in reaching agreements, including the group's power, quorum requirement, and any external influences. |
| **Expected Outputs** | - List the planned project outputs and deliverables, including timelines and milestones, if applicable. - Include the opportunities (e.g. co-authorship, presenter) provided to the members for each expected output. |
| **Recognition of Contributions and Potential Benefits** | - List all compensations (e.g. honoraria and/or reimbursement) and methods (e.g. publication acknowledgements) for recognizing member contributions and participation, including honoraria and reimbursement, and highlight potential benefits to motivate participation. - Discuss with members on preferred ways of recognition and additional opportunities to participate in engagement activities not initially listed in the research plan. |
| **Evaluation** | - The evaluation section outlines the process of measuring the engagement process's impact on the study, panel members, and the ToR as a tool for engagement. |
| **Include any applicable references and resources within any of the sections.** | |

**Appendix II. Feedback Checklist for Terms of Reference**

| **Important Tips for Obtaining Feedback** |
| --- |
| - Provide a simple process for obtaining feedback from knowledge users. |
| - Schedule milestones and check-in points to obtain feedback from knowledge users. |
| - Include prompting questions and discussion points to facilitate reflection from knowledge users. |
| - Ensure enough time is given and be flexible in the deadlines provided. |
| - Create closed-looped and iterative systems for changing and implementing the ToR by sharing all updates, outcomes and impacts from the document. |
| - Be flexible in the mode of receiving feedback from knowledge users to accommodate varying preferences. |
